# Ultrasound-Assisted Management for Tracheal Intubation in the Patient with Tracheal Diverticulum

**DOI:** 10.1155/2023/5586490

**Published:** 2023-09-19

**Authors:** Tingting Wan, Ya Gao, Changyi Wu

**Affiliations:** Department of Anesthesiology, Peking University Third Hospital, Beijing 100191, China

## Abstract

Tracheal diverticulum (TD) is a rare disease. Due to the worldwide pandemic of COVID-19, the increase of routine preoperative chest CT examination has led to a higher detection rate of TD. Although TD is very rare, it is one of the reasons for difficult intubation and difficult ventilation. Improper treatment can cause severe airway emergencies such as diverticulum tearing, tracheal rupture, and subcutaneous or mediastinal emphysema. Unfortunately, there are few studies on TD, especially in perioperative airway and anesthesia management. This paper reports a case of TD found by preoperative chest CT examination who required tracheal intubation under general anesthesia. For the first time, ultrasound was used to confirm the position of tracheal tube and TD, and good results were achieved. This attempt provides a new idea and method for airway management in patients with TD.

## 1. Introduction

Tracheal diverticulum (TD) is a benign cyst from the posterior wall of the trachea, most commonly seen in the right posterior side of the trachea and at the level of T2 or T3 [1, 2]. The disease is rare, with a prevalence rate of 1%–3.7% [3]. TD is usually found by fiberoptic bronchoscopy or neck and chest CT occasionally. A small number of patients were treated for cough, recurrent respiratory tract infection, hoarseness, or dyspnea [3, 4]. Although TD is very rare, it is one of the reasons for difficult intubation and difficult ventilation. Improper treatment can cause severe airway emergencies such as diverticulum tearing, tracheal rupture, and subcutaneous or mediastinal emphysema [5, 6]. Unfortunately, there are few studies on TD, especially in perioperative airway and anesthesia management. This paper reports a case of TD found by preoperative chest CT examination who required tracheal intubation under general anesthesia. For the first time, ultrasound was used to confirm the position of tracheal tube and TD, and good results were achieved.

## 2. Case Presentation

**Figure 1:**
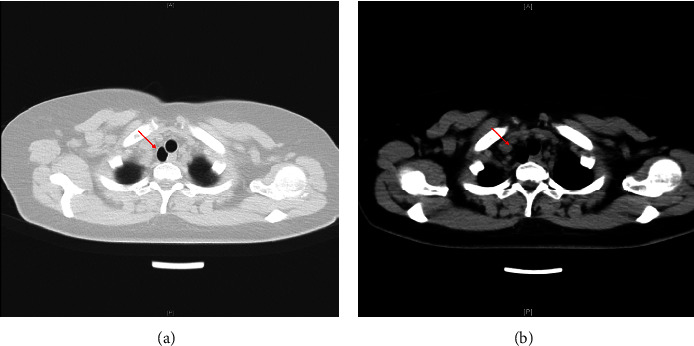
Chest CT cross-sectional images of lung window (a) and mediastinal window (b) (the arrow indicates the TD).

A 59-year-old female (BMI 26.72 kg/m^2^) was diagnosed with right thyroid nodules by ultrasound for 3 years. The patients had no symptoms such as neck pain, hoarseness, choking or coughing while drinking water, dysphagia, dyspnea, palpitations, or emotional irritability. Thyroid ultrasound showed multiple nodules in bilateral thyroid lobes. The maximum nodule on the left side was 0.5 cm × 0.3 cm × 0.3 cm, and the maximum nodule on the right side was 1.8 cm × 1.2 cm × 1.1 cm. Preoperative chest CT showed cystic shadow in the right rear of the trachea (2.2 cm × 1.4 cm × 1.7 cm in size) and an opening in the right rear of the trachea in the mediastinal window, which was diagnosed as TD (Figure 1). There was no abnormality in laboratory tests and other auxiliary examinations. A total right thyroidectomy was scheduled under general anesthesia, and the histopathology of the nodules was detected during the operation.

**Figure 2:**
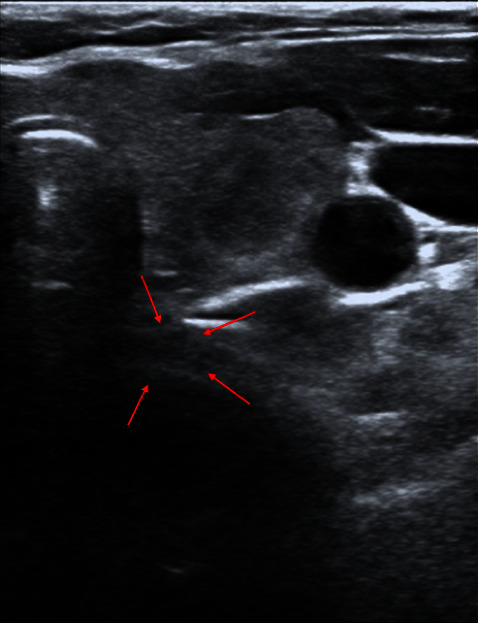
Right rear TD in spontaneous breathing.

**Figure 3:**
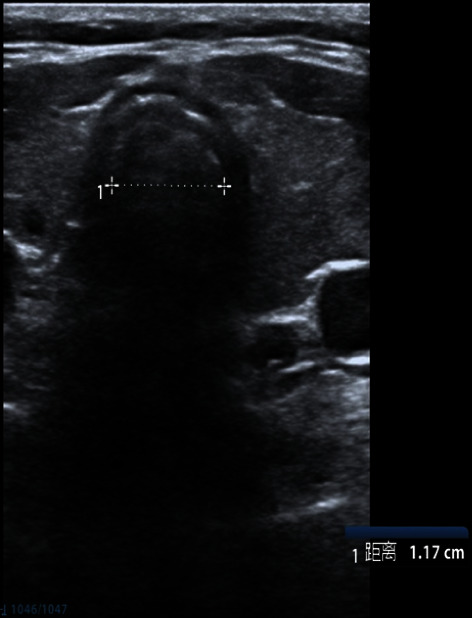
Measure the inner diameter of the main trachea.

**Figure 4:**
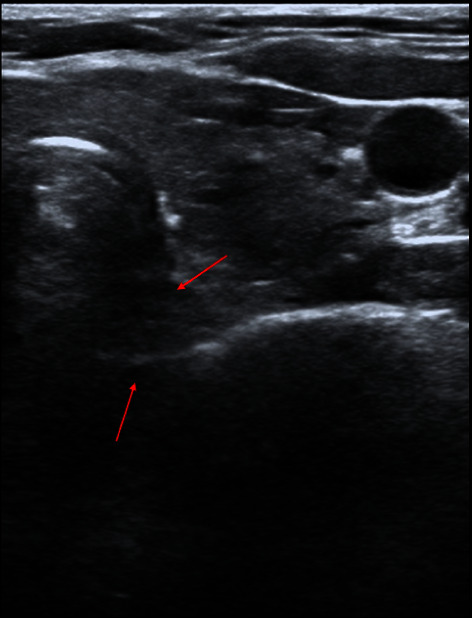
Image of tracheal tube water sac and TD in the same plane.

The patient had fasted for 8 hours before surgery. ECG, SpO_2_ and noninvasive blood pressure were monitored routinely after arrival in the operating room. The right posterior TD was seen under ultrasound (Figure 2), and the airway diameter was measured (Figure 3). After determining the position of diverticulum, general anesthesia induction was performed. The patient inspired 100% oxygen at 6 L·min^−1^ through a transparent facemask for 3 minutes. Then, etomidate (12 mg), sufentanil (20 *μ*g), lidocaine (40 mg), and cisatracurium (12 mg) were intravenously injected in turn. Propofol (40 mg) was added each time according to the circulation changes. Artificial ventilation was performed after the disappearance of consciousness. Under ultrasound, it can be observed that the TD expanded with positive pressure ventilation during inspiration and had an obvious opening, and the TD shrinked after chest rebound (video 1, https://v.youku.com/v_show/id_XNTkxNDQ0NjkwNA==.html). After artificially-assisted ventilation for 5 mins, fiberoptic bronchoscopy was performed, and no TD opening was observed. Then, reinforced tracheal tube (no. 7.0) was placed under the guidance of fiberoptic bronchoscopy. 5 ml normal saline was injected into the tracheal tube cuff, and the position of the water sac and the TD was adjusted under ultrasound (Figure 4). After confirming that the tracheal tube cuff was located distal to the TD opening, the tracheal tube was placed at a depth of 24 cm. The respiratory sounds of both lungs were symmetrical, and the tracheal catheter was properly fixed. A low tidal volume strategy was used (tidal volume was 6 mL/kg and respiration rate was adjusted to maintain end-tidal CO_2_ at 35–40 mmHg). The thyroid was carefully separated during the operation. After the right thyroid was removed, the right posterior TD was not observed. The operation lasted 55 mins and went uneventfully. After operation, the patient was extubated normally after being awake, and there was no obvious discomfort.

## 3. Discussion

Currently, due to the worldwide pandemic of COVID-19, the increase of routine preoperative chest CT examination has led to a higher detection rate of TD. There are two different types of tracheal diverticulum: congenital and acquired. Congenital TD is a cystic structure connected to the trachea through a narrow isthmus, and the opening may sometimes be invisible. However, acquired TD is a cystic structure in the weak part of the tracheal wall due to the increase of intraluminal pressure. Acquired TD has larger opening and wider connection with trachea than congenital TD, which is a major cause of complicated orotracheal intubation [7]. The patient in this case usually has no related clinical manifestations of respiratory system, and it is considered to be a high possibility of congenital TD.

Although the incidence of TD is low, anesthesiologists should fully evaluate the airway management to avoid adverse consequences due to improper handling. Unfortunately, there is no unified standard for the airway management of patients with TD at present. Most tracheal intubations are guided by fiberoptic bronchoscopy with the tip or distal end of the tracheal tube over the TD opening to avoid TD rupture or perforation due to the change of surgical position or excessive ventilation pressure [8, 9]. The tracheal cuff should avoid getting stuck at the TD opening to prevent iatrogenic TD [10] or tracheal rupture due to excessive pressure. One case reported the tracheal cuff leakage caused by the TD opening. The problem was solved by replacing a larger diameter tracheal catheter and locating the distal catheter above the diverticulum opening [11].

In recent years, ultrasound technology has been widely used in airway and respiratory system. Ultrasound can be used to predict the difficulty of endotracheal intubation, determine the inner diameter of the airway, and predict the appropriate size of single-lumen and double-lumen endotracheal intubation and tracheostomy catheters. Ultrasound can also be used to diagnose vocal cord paralysis or tracheal stenosis, accurately locate the cricothyroid membrane for emergency thyrocricoid puncture, identify tracheal ring for ultrasound-guided tracheostomy, etc., [12–14]. In this case, the opening of the TD was not detected by fiberoptic bronchoscopy, but the opening was visible on ultrasound because positive pressure ventilation was performed, which caused gas to open the TD opening. Therefore, this case was the first attempt to apply ultrasound technique to the airway management of TD. Compared to fiberoptic bronchoscopy, it has unique advantages. Of course, this method also has certain limitations. The TD below the level of the suprasternal notch will not be detected by ultrasound. In addition, there are limitations for patients without preoperative CT scan because it is difficult to make direct judgment of TD only by ultrasonic examination. However, with the pandemic of COVID-19 in the world, a lot of patients undergo CT scan preoperatively, and more patients with TD have been diagnosed. So, the application of ultrasound provides a new idea and method for airway management in patients with TD.

## Data Availability

The data used to support the findings of this study are included within the article.
